# Patients' Attitudes towards the Surplus Frozen Embryos in China

**DOI:** 10.1155/2013/934567

**Published:** 2012-12-26

**Authors:** Xuan Jin, GongXian Wang, SiSun Liu, Ming Liu, Jing Zhang, YuFa Shi

**Affiliations:** ^1^Centre for Assisted Reproduction, The First Affiliated Hospital of Nanchang University, Jiangxi, Nanchang 330006, China; ^2^Department of Urology, The First Affiliated Hospital of Nanchang University, Jiangxi, Nanchang 330006, China; ^3^Department of Obstetrics and Gynaecology, The First Affiliated Hospital of Nanchang University, Jiangxi, Nanchang 330006, China

## Abstract

*Background*. Assisted reproductive techniques have been used in China for more than 20 years. This study investigates the attitudes of surplus embryo holders towards embryos storage and donation for medical research. *Methods*. A total of 363 couples who had completed in vitro fertilization (IVF) treatment and had already had biological children but who still had frozen embryos in storage were invited to participate. Interviews were conducted by clinics in a narrative style. *Results*. Family size was the major reason for participants' (dis)continuation of embryo storage; moreover, the moral status of embryos was an important factor for couples choosing embryo storage, while the storage fee was an important factor for couples choosing embryo disposal. Most couples discontinued the storage of their embryos once their children were older than 3 years. In our study, 58.8% of the couples preferred to dispose of surplus embryos rather than donate them to research, citing a lack of information and distrust in science as significant reasons for their decision. *Conclusions*. Interviews regarding frozen embryos, including patients' expectations for embryo storage and information to assist them with decisions regarding embryo disposal, are beneficial for policies addressing embryo disposition and embryo donation in China.

## 1. Introduction

According to a 2006 report by the International Committee Monitoring Assisted Reproductive Technology, more than 3 million babies worldwide were conceived through in vitro fertilization (IVF) or intracytoplasmic sperm injection (ICSI) [1]. The development of advanced techniques to cryopreserve embryos has made the cryopreservation of surplus embryos an integral part of IVF procedures, as a result improving pregnancy rates and reducing risks and costs. However, the number of embryos in storage around the world is steadily increasing, to the point that the increasing number of embryos in storage has become a burden for the centre of assisted reproduction. In some countries, legislation stipulating a maximum storage period of human embryos has come into effect. Storage limits vary between countries and between the states within some countries; most countries have determined that embryos can remain in storage for up to 5 years [2–9]. Such legislation prevents an unlimited accumulation of stored embryos. When surplus embryos approach the storage time limit, there are several options for patients. For example, in Australia, the embryos can be destroyed, donated to infertile couples, or donated to science (since 2003, following the enactment of federal legislation permitting embryonic research under some conditions) [4]. These three options also exist in the United States of America, following the 2010 removal of the ban on the use of federal funds for the creation or use of new cell lines generated from embryos [10]. In Switzerland, surplus embryos can either be disposed of or used for medical research under certain restricted conditions [2].

In China, since 1988 when the first baby was born by IVF, millions of infertile couples have had opportunities to conceive and give birth to their own children; by thawing and transferring cryopreserved embryos, patients who undergo IVF or ICSI have greater chances of becoming pregnant without undergoing new stimulation cycles. The legislation and guidelines in China specify that (1) two embryos can be transferred at the same time in patients under 35 years old and in their first stimulation cycles; (2) three embryos can be transferred at same time in patients aged 35 years or older, or in patients younger than 35 years old but in their second stimulation cycles; (3) three frozen embryos can be transferred at the same time to a patient of any age, unless it is the first embryo transfer and she is younger than 35 years old, in which case two frozen embryos should be transferred [11, 12]. The surplus embryos that are not transferred are stored and used in subsequent treatment cycles to enable pregnancy. As a result, the number of frozen embryos accumulates constantly; the most difficult problems associated with a long duration of embryo cryopreservation are the increase of “unclaimed” embryos and the associated ethical, legal, and economic pressures. All of these problems require the centre of assisted reproduction to determine the fates of surplus frozen embryos. In China, no law has been adapted specifying a maximum storage limit of cryopreserved human embryos [11, 12]. This study is the first in China that assesses attitudes towards the disposition of surplus embryos and, involves a large representative sample of couples who already have children and still have cryopreserved embryos. The study includes two sections that (1) investigate attitudes towards the discontinuation of surplus embryo storage and (2) investigate attitudes towards the donation of surplus embryos for research.

## 2. Materials and Methods

### 2.1. Ethics and Recruitment

In China, IVF treatments can only be provided by licensed centres. This study was approved by the Human Research Ethics Committee at the First Affiliated Hospital of Nanchang University. Before cryopreservation, all patients at the IVF centre at the first affiliated hospital of Nanchang University signed a form allowing medical staff to freeze and store their supernumerary embryos (if the quality of these embryos was considered sufficient for cryopreservation). 

Participants were recruited from the IVF centre at the first affiliated hospital of Nanchang University. All participants had biological children and still had surplus embryos stored at the centre; couples who did not have surplus embryos in storage or who had surplus embryos in storage but did not have biological children were excluded from the study. At the beginning of the procedure, 427 couples who had completed IVF treatment between 2001 and 2011 were included; the actual number of couples invited to the interview was 363 (85%). In 59 cases, the patients could not be reached due to changes in their contact information (that the clinic was not notified of), and in the other 5 cases, the patients refused the interviews. About 63% of the participants were living in Jiangxi province, while the rest lived in other provinces. On the basis of their children's ages, we separated the participants into three groups: 0–3 years old (group I), 3–5 years old (group II), and older than 5 years (group III). 

### 2.2. Procedure

All in-depth interviews (in a narrative style) were conducted at the IVF centre by the researchers, either in person or by telephone, between January and April of 2012. Each interview was about 0.5 hours in duration. With the participants' permissions, the personal interviews were recorded and transcribed by an independent transcriber and checked for accuracy. All the names for persons were replaced by numbers (i.e., patient number 1). The interviews had two purposes: (1) to collect a range of participant decisions regarding the destinations of their surplus embryos and (2) to collect a range of information in relation to their decisions on the excess embryos, including the reasons and feelings behind their decisions, as well as the educational backgrounds of the participants. 

An interview guide was used, couples were interviewed together. First, participants were asked what decisions they had made regarding their excess embryos (continuing or discontinuing storage). Second, the participants who chose to discontinue the storage of their embryos were asked about their thoughts regarding the fates of their unused embryos, which would be either donated to research or disposed of. Finally, the participants were asked to describe their reasons for choosing their preferred options and how they felt about the decision-making process. 

It is worth noting that because no legislation permitting the use of surplus cryopreserved embryos for research has thus far come into effect in China, the discussion about donation to research was in response to a hypothetical scenario, rather than actual practice. 

### 2.3. Analysis

The data were analysed using SPSS software (version 11.5). Associations were assessed using chi-square test; *P* values of <0.05 were considered to be statistically significant. 

## 3. Results

### 3.1. Attitudes towards the Storage of Cryopreserved Embryos

Although there is one-child policy in China presently, storage of embryos is permitted in case an accident happened to the child and parent still have chance to get another child if their only one child was dead or had some disease which is under one more children born permission. Besides of that, to ensure the possibility of pregnancy, two embryos would be generally transferred into uterus each time, which caused the rate of twins higher by IVF than that by natural fertility.  Table 1 shows couples' attitudes towards the storage of their cryopreserved embryos and towards the continuation or discontinuation of storage. In the group with children aged 0 to 3 years old (group I), 83.3% of the couples choose to continue storing their embryos, only 16.7% would discontinue their embryos' storage, and one person declined the interview. In the group with children aged 3 to 5 years old (group II), among the 131 participants, 26.7% wanted to continue storing their embryos; 73.3% discontinued storing their embryos, and two people declined the interview. In the group with children older than 5 years (group III), 25% wanted to continue storing their embryos, and 75% discontinued; this group was similar to group II. There were no differences in terms of attitudes towards the storage of embryos between the provincial regions (data not shown in table). 

### 3.2. Reasons to (Dis)continue Storage of Surplus Embryos

#### 3.2.1. Continue Storage

As Table 2 shows, wanting more children was the major reason cited by most of the couples who wanted to continue storing their embryos; in the three groups, the percentages reporting this reason were 83.3%, 74.2%, and 100%.

*Patient Number 1. We plan to have more babies in three years, so we want to continue the embryo storage for 3 years; maybe next time, we will have a boy.*

*Patient Number 2. I know only one child is permitted per couple by Chinese law, but I still hope that maybe one day more children will be permitted in China; then, we will have a chance to have more children. *

*Patient Number 3. We are from the countryside, so we should have a boy.*

Worrying about their children's health was another important reason reported within groups I and II, whose children were younger than 5 years old. This fear decreased when the children were older than 5 years; only 30% of participants worried about their children's health at that point, compared with about 50% in groups I and II.

*Patient Number 4. Please keep storing our embryos, just in case; our baby is so young, after all, and we are not sure what will happen to him. *

In addition, the moral status of embryos was a reason named by couples who wanted to continue storing their embryos. In these cases, the storage decision was not always consistent with a desire for more children. However, few storage decisions were linked to religion(datanot shown).

*Patient Number 5. The embryos are still life. Although I have not made a decision about whether I will transfer them or not, they are still potential life.*

*Patient Number 6. Yeah, they are our children who have not been born; we have tried so hard to have the embryos, so we will continue storing them.*

*Patient Number 7. It is so hard for us to make this decision. Can we postpone the decision making? If not, we choose to continue the storage.*

#### 3.2.2. Discontinue Storage

Among the reasons that couples chose not to store their embryos, family completion and storage fees were two common reasons.

*Patient Number 8. The storage fee is a big financial burden for us. Since we have a baby, we won't pay the fee to continue the storage of the embryos.*

*Patient Number 9. We have two children from the last IVF treatment. This is enough for our family; we do not want more children, so the surplus embryos need not be stored anymore.*

Concern about the quality of frozen embryos increased along with the duration of embryo storage; this was an important reason for the couples in group III who did not want to continue storage (50%, compared with 10.4% of group II and 0% of group I). 

Five women achieved natural pregnancy; they all chose to discontinue storage. 

### 3.3. Desire for Extra Embryos Storage

Table 3 shows the differences in men's and women's desires for surplus embryo storage. In group I, both the husbands and wives had strong desire to continue storing the surplus embryos. As the children got older, however, the men's desire to store embryos decreased correspondingly. In group III, when the children were older than 5 years, 75% of the embryo storage decisions came from the women alone, in contrast to 100% among the couples in group I.

*Husband of Patient Number 5. About the destination of our frozen embryos, I do not care. It depends on my wife's attitude; whatever decision she makes, I agree.*

### 3.4. Attitudes towards the Destination of Surplus Embryos

Table 4 shows the couples' attitudes towards the destinations of surplus embryos that they discontinue storing. More people were likely to discard their embryos than to donate them to research or therapy (58.8% versus 41.2%, resp.). In the approximately two-fifths of participants who supported embryo donations, about 28.7% considered donating to medical therapy (if available) but did not want to donate to medical research.

*Patient Number 10. By donating the embryos to medical therapy, I felt they might save somebody. But with research, I cannot imagine what kind of research is done on the embryos; after all, they are like my unborn children.*

Among all of the correlates entered into the model predicting couples' attitudes towards donation to medical research or therapy and disposal, educational level had no relationship with the decision regarding the destination of the embryos; of the participants, 68.1% of high school graduates and 31.9% of college and university graduates preferred donation, while 69.4% of high school graduates and 30.6% of college and university graduates chose to discard the embryos. The differences in couples' views on the moral status of embryos had a significant impact on the decision to discard or donate embryos; about 45.5% of the couples who choose to discard their embryos felt that an embryo is just a cluster of cells, while the 13.6% of couples choosing donation considered the embryo to be life or a potential child. For these couples, the donation decision was difficult, and they hesitated.

*Patient Number 11. No, I disagree with donation. Actually, I care about how the embryos are disposed of. You know, they deserve at least some sort of respect.*

*Patient Number 12. No, I cannot imagine donating the embryos to research. I would donate my own body or organ when I die if it is needed, but donating my embryos, I cannot accept that.*

*Patient Number 13. Yes, I prefer donation of the embryos to medical research or therapy. Since my family is complete now, we will be happy that they could help others.*

*Patient Number 14. The embryos are our potential children. I disagree with donation.*

Two hours after the end of the interview, patient Number 14 called the investigator, saying the following.

*Patient Number 14. We, as a couple, discussed donation again. We prefer to donate our embryos to medical research or therapy; we hope that they can help others.*

## 4. Discussion

This study consists of a first survey conducted in China using a relatively large representative sample of patients who have either experienced successful IVF/ICSI treatment or experienced natural pregnancy later but still owned cryopreserved embryos. Cryopreservation of embryos for later use is a routine procedure in China, as no strict legal regulations or deadlines for the destruction of cryopreserved embryos preserved for reproductive purposes have thus far been adopted. Maintaining the accumulating “unclaimed” embryos in infertility centres is an expensive and time-consuming task; this has become a common problem. In this paper, we address the influence and relevance of the external context, such as embryo holders' expectations for and perceptions of frozen embryos and storage fees. 

Our survey showed that couples' storage decisions largely depended upon the ages of their children. When the children were aged between 0 and 3 years, more than four-fifths of the couples wanted to maintain their embryos; of those, some couples were not sure whether they would transfer the embryos. Conversely, when their children grew older (older than 3 years), 70% of the couples chose to discontinue the storage of their embryos. Of the reasons behind the storage decisions, the desire to have more children was the most important factor, as has been described in other studies [3, 13, 14]. Additionally, worrying about the children's health was a concern for couples when their children were younger than 5 years old. Another type of storage decision, aside from the desire for more children, concerned the moral status of embryos; this emotional reason, despite the absence, in some cases, of another purpose, could be a factor adding to the number of participants who were hesitant and wanted to postpone their decision making as long as possible. This finding is consistent with other studies [5, 15, 16]. No significant regional differences based on the reasons for storage were found.

We found that participants' attitudes towards storage fees generally varied with their stages of decision making. Most of them told us that without storage fees, they would have little motivation to make a disposal decision and would instead keep the embryos in storage indefinitely. Storage fees were one reason that some participants abandoned frozen embryos, which cannot be neglected [17–22]; this was especially true among residents from the countryside. 

Family size was also found to be an important determinant. Because of the one-child policy in China presently, in our sample, couples who already had an average of two children were generally satisfied with their family sizes and felt that their families were complete. Other participants, however, reportedly would consider using surplus embryos in the future when the one-child policy changes. The importance of family size has also been found in other studies [5, 13, 23]. Besides of the family size, the preference for male children is one more reason for the couples to keep their embryos storage. In China, bearing progeny is generally considered as part of a stable marital nexus. Children, particularly sons, are regarded as a continuance of a whole family. Furthermore, sons are regarded as a source of income and security in old age for many lower income or lower educated people. The third reasonable one that the couples would consider is also the effect of China's one-child policy on aged couples. There are some aged couples, their only child died for some reason, they had to suffer the lonely life without children at the rest of their time, because wive's ovary is failure and no available oocytes can be used at that time. That is why some couples continue their embryos stored, just in case. 

Another interesting finding in our survey was that the duration of embryo storage was strongly linked to patients' choices for their embryos' fates; nearly half of the participants opted to abandon their surplus embryos once they had been stored for more than 5 years, as they were not confident in the quality and integrity of their cryopreserved embryos. 

In terms of attitudes towards embryo storage, women tended to be more preoccupied with the statuses of their embryos than their husbands were. In couples who did not desire more children, it was more difficult for the women to make decisions about the fates of their surplus embryos; deciding to dispose of their embryos was always emotionally fraught for them. In addition, women's opinions regarding excess embryos were usually respected by their partners. The moral status of embryos plays an instrumental role in the symbolic meaning of the relationship between partners [16]. 

Donation to science is another option for the disposal of surplus embryos. Recent observations have shown that donation to medical research or therapy has become more popular; some participants who would probably donate to research described discarding embryos as wasteful or selfish [24–26]. Our findings are not entirely consistent with published reports; in fact, 41.2% of couples chose donation to research, versus 58.8% who preferred to discard their excess cryopreserved embryos. The decision not to donate was, for many couples, linked to their relationships with the embryos. The participants' views of embryos as potential life have direct implications with respect to their views of donation of embryos for stem cell research, which is consistent with a previous study [24]. For most of the individuals interested in donation, embryos were viewed as a cluster of cells. 

In addition, we found that lacking information about what the research using embryos actually entailed was another significant factor for the patients who were more likely to discard their embryos. Because of the absence of specific information, the participants thought that discarding their embryos would prevent them from being misused. A minority of couples stated they would donate to medical therapy, but not to research. This distrust in science or scientists consequently affected donation decisions, as was also described in earlier papers [17, 18, 25, 27]. Moreover, the educational backgrounds of participants did not seem related to their decisions. A survey in the US reported that Asians who immigrated to the US were less likely to donate excess cryopreserved embryos for research use than Caucasians and Asians born in the US and that they were more likely to discard embryos; embryo disposition plans among Asians born in the US, on the other hand, were similar to those of all other ethnicities [28]. With more permissive regulations than those in the US, between 2001 and 2009, human embryonic stem cell research has been performed on a large-scale in China, Japan, South Korea, Singapore, India, and Taiwan [29]; thus, cultural bias against human embryonic research is unlikely a real reason. Many interviews suggested that acquainting participants with more information about the kinds of research being done or directing them to a specified research project would assist them in choosing a donation option. The feeling that parents still have some control over the type of research project for which their embryos would be used is definitely influential. Therefore, our study suggests that misunderstanding or not receiving adequate information about the disposal choices is a significant reason for many stem cell candidates to refuse donation; this conclusion is consistent with previous reports [30, 31]. It is interesting to note that 92% of couples preferred to donate their supernumerary embryos to stem cell research instead of discard them, according to a Swedish study; furthermore, the high level of interest in donation is due partly to the detailed and comprehensive oral and written information about the specific research project that is given to each patient [32]. In addition to adequate information, additional psychological support is also helpful, particularly for couples hesitating to donate to research. A few couples admitted that the decision to discard their embryos was made at the “last minute.” In the US, studies performed in the 1990s indicated little willingness to donate embryos to science [33–35]. In contrast, donation to science has become an increasingly popular disposal option in recent years [24–26]. This apparent shift in patient attitudes may reflect increased public awareness of stem cell research in the U.S. 

Another interesting finding from our survey is that religious consideration did not play a significant role in couples' storage decisions or donations to research. The influence of religious beliefs has yielded inconsistent results in previous studies [2, 4, 16, 36]. 

## 5. Conclusion

On the whole, maintaining large numbers of frozen embryos is currently quite a burden for IVF centres in China. In some countries, legislation limiting the amount of time that embryos can be stored has been established, resulting in large scale disposal of abandoned embryos [2–9]. In China, the one-child policy and its effect on couples' family planning decisions should also be considered to the rule of embryos storage setting up. Another strategy that we recommend to IVF clinics around the world is providing more detailed information regarding research, directed research options, and even psychological support groups to assist in decision making by embryo holders. Although it is a complex issue, it is still worth considering. 

## Figures and Tables

**Table 1:** The participants' attitudes towards the storage of their cryopreserved embryos.

Total participants	Respondents	Continue storage		Discontinue storage		Reject
*N*: 363	*n*	*n*	%	*n*	%	*n*
Group I Children 0–3 years old	72	60	83.3	12	16.7	1
Group II Children 3–5 years old	131	35	26.7	96	73.3	2
Group III Children >5 years old	160	40	25	120	75	2

**Table 2:** Participants' reasons to (dis)continue storage.

	Group I Children 0–3 years old		Group II Children 3–5 years old		Group III Children >5 years old	
Reasons to continue storage	*n* = 60		*n* = 35		*n* = 40	
	*n*	%	*n*	%	*n*	%

Worrying about the children health	30	50	17	48.6	12	30
More baby wish	50	83.3	26	74.2	40	100
Embryo is potential life	20	33.3	17	48.6	20	50

Reasons to discontinue storage	*n* = 12		*n* = 96		*n* = 120	
	*n*	%	*n*	%	*n*	%

Storage fee	12	100	57	59.3	60	50
Family is complete	12	100	86	89.6	80	66.7
Achieved natural pregnancy	0		5	5.2	0	
Worrying about frozen embryos quality	0		10	10.4	60	50

**Table 3:** Difference of desire for extra embryos storage.

	Group I (children 0–3 years old)		Group II (children 3–5 years old)		Group III (children >5 years old)	
	*n* = 60		*n* = 35		*n* = 40	
	*n*	%	*n*	%	*n*	%
Women's desire	0		10	28.6	30	75
Men's desire	0		12	34.3	5	12.5
Couples' desire	60	100	13	37.1	5	12.5

**Table 4:** Attitudes toward destination of surplus embryos and correlates associated with the decision.

Discontinue storage of embryos *n* = 228	Donation						Discarding	
			*n*	%			*n*	%
			94	41.2			134	58.8
	To medical research only		To medical therapy only		To medical research and therapy			
	*n*	%	*n*	%	*n*	%		
	0		27	28.7	67	71.3		
Correlates associated with couples' decision								
			*n*	%			*n*	%

Education								
Elementary school			0				0	
High school			64	68.1			93	69.4
College and university			30	31.9			41	30.6
Participants' view of the moral status of embryos								
Cluster of cells			81	86.2			61	45.5
Life, potential child			13	13.6			73	55.5
